# In this month’s Bulletin 

**DOI:** 10.2471/BLT.23.000723

**Published:** 2023-07-01

In the editorial section, Irene Papanicolas et al. (438) call for papers for an upcoming theme issue on health system performance assessment. 

Gary Humphreys (441 – 442) reports on innovative approaches to vector control in the Region of the Americas and speaks to Jackeline Alger (443 –444) about building public health research capacity in Honduras against a backdrop of conflict and political upheaval.

## China

### Targets for dietary sodium reductions

Puhong Zhang et al. (453 – 469) quantify sodium levels in pre-packaged foods. 

## India

### COVID-19 and tuberculosis 

Keertana Duppala et al. (445 – 452) test feasibility of simultaneous screening. 

## Mongolia 

### Unintended effects of a raw coal ban 

Tsetsegee Sambuu et al. (470 – 477) detect an increase in domestic cases of carbon monoxide poisoning. 

## Rwanda

### Early detection of breast and cervical cancers

Lydia E Pace et al. (478 – 486) trial combining clinical breast examinations with cervical cancer screening.

## Viet Nam

### Artificial intelligence and dengue surveillance

Ho Quang Chanh et al. (487 – 492) share lessons learnt with a prototype wearable device. 

